# Mixed-Immigration Status Families During the COVID-19 Pandemic

**DOI:** 10.1089/heq.2022.0141

**Published:** 2023-04-20

**Authors:** Stephanie Iraheta, Brittany N. Morey

**Affiliations:** Department of Health, Society, and Behavior, Program in Public Health, University of California, Irvine, Irvine, California, USA.

**Keywords:** mixed-status families, COVID-19, immigration policy

## Abstract

**Introduction:**

To understand how mixed-immigration status families—families with a mixture of people with and without documentation—in the United States (U.S.) fared during the COVID-19 pandemic. Specifically, this study highlights how health inequities were exacerbated during the height of the pandemic due to the implementation of anti-immigration policies such as Public Charge Rule, which stipulates that receiving public benefits is grounds for inadmissibility for immigrants seeking naturalization.

**Methods:**

In-depth semistructured interviews were conducted over Zoom with 14 members of mixed-status families between February and April 2021. The interviews were audio recorded, transcribed, and analyzed using Atlas.ti. Using grounded theory, we assessed the level of awareness about Public Charge Rule and the health challenges these families faced during the COVID-19 pandemic.

**Results:**

Themes that emerged included financial problems, job insecurity, housing insecurity, food insecurity, mental health problems, distrust of government and health officials, and a fear of Public Charge Rule. We present a framework for understanding health inequities for mixed-status families during the COVID-19 pandemic.

**Discussion:**

Public Charge Rule caused fear and confusion for mixed-status families during the COVID-19 pandemic, resulting in individuals not receiving public benefits they urgently needed. This created heightened mental health problems due to job, housing, and food insecurity.

**Health Equity Implications:**

We discuss how trust between mixed-status families and the government needs foundational rebuilding. In addition to streamlining the process for these families to apply for legal status, it is important to protect and support mixed-status families through programs and policies during public health emergencies.

## Introduction

The COVID-19 pandemic has laid bare disparities for the most socially disadvantaged communities in the United States (U.S.), including those disadvantaged due to immigration and documentation status.^1–5^ This was especially true for mixed-status families, who were often overlooked during the pandemic. These are families that consist of a mix of undocumented residents, legal immigrants, and/or American citizens—with many of the latter, including the U.S.-born children of undocumented parents.^6^ In the nation as of 2016, about 20.2 million people lived in households with at least one undocumented person, and of these, 9.5 million are U.S.-born minor or adult children.^7^ While these families qualify to receive government support due to the presence of U.S. citizen family members, the parents were hesitant to receive any support on their children's behalf.

The goal of this research is to examine how fears of repercussions from anti-immigration policies affected mixed-status families' health and access to desperately needed resources during the COVID-19 pandemic.

Families' decisions on whether to receive support have been impacted by anti-immigration policies such as zero tolerance policies and Public Charge Rule.^8–10^ Public Charge Rule is a policy stating that receiving public benefits would be grounds for inadmissibility for immigrants seeking naturalization through the legal process.^11^ Public Charge Rule was approved by the Trump Presidential Administration in August 2019, and the U.S. Department of Homeland Security began implementing the rule on February 24, 2020,^12^ just before the World Health Organization declaring COVID-19 a pandemic in March 2020. A report published by the Urban Institute revealed that the threat of Public Charge increased fear among immigrant families regarding accessing benefits or assistance for themselves or their children due to the possibility of doing so leading to deportation or jeopardizing the legal process for naturalization.^13^

During the COVID-19 pandemic, Public Charge Rule was slightly eased for extreme cases of need deemed credible by the federal government.^8^ However, this easement of the Public Charge Rule was not advertised widely, and undocumented individuals likely still feared receiving any help from the government during the public health emergency.^14^

Although the U.S. federal government allocated resources to help families during the COVID-19 pandemic, mixed-status families were either ineligible or fearful of receiving needed help. Federal health benefits such as the Affordable Care Act excludes undocumented individuals, forcing them to pay for medical care out of pocket.^15^ Locally in places such as Los Angeles County, there are programs that offer health care for the undocumented community, but health care providers report that they suspect many individuals do not seek these services due to Public Charge Rule.^15^ The Coronavirus Aid, Relief, and Economic Security (CARES) Act passed in March 2020—which provided economic relief, increased unemployment benefits, and direct cash payments to individuals—also excluded undocumented people.^16^ With the lack of financial support, undocumented individuals suffered economically and likely further avoided health care due to the expense, even if infected with COVID-19.^3,11^

While the CARES Act was updated in late December 2020 to include some mixed-status families during the second round of stimulus checks, those with two undocumented parents and U.S. citizen children still were not included within the package.^17^ To qualify, one household member needed a social security number, which was not the case for many mixed-status families. Lacking support from governmental and public benefits likely exacerbated health disparities, worsening the impact of COVID-19 on the undocumented community.^18^

Current studies emphasize how people marginalized at the intersections of race, socioeconomic status, and immigration status have been affected by the COVID-19 pandemic.^5,19^ This article seeks to fill a major gap in research on mixed-status families, to understand how they were affected by COVID-19 and how they navigated accessing resources in an anti-immigration policy environment. Using qualitative methods, this research aims to answer two major questions: (1) How did the anti-immigrant sociopolitical environment, including fear of Public Charge Rule, impact mixed-status families' help-seeking behavior during the COVID-19 pandemic?^20^ and (2) How did these decisions affect mixed-status families' mental health, financial, job, housing, and food insecurity?^21,22^ Understanding the impact of COVID-19 on mixed-status families will help to develop more equitable policies and programs to increase resiliency for these contributing members of American society.^23,24^

## Methods

This study consisted of semistructured qualitative interviews with adult members of mixed-status families conducted by the lead researcher between February and April 2021. Online recruitment consisted of convenience sampling through digital flyers distributed on Facebook, Instagram, and Twitter. Flyers were also posted on newsletters of the Cross-Cultural Center, Latinx Resource Center, and Dream Center for the University of California, Irvine (UCI). UCI has a relatively large population of students from mixed-status families. Participants were 18 years or older, part of an immediate mixed-status family, and residents of the Southern California region. Recruitment was conducted in both Spanish and English. All participants provided informed consent and received a $15 Amazon gift card. The research protocol was approved by the Institutional Review Board of UCI. A total of 14 individuals from mixed-status families participated.

Interviews were conducted over Zoom for 45-min with each participant. Interviews were audio recorded and were conducted in Spanish or English, according to participants' preferences. The interview consisted of general introduction questions that included the collection of the documented statuses of participants and their family members, as well as information such as age, employment, and breadwinner status (whether they were the primary household income producer). The second portion of the semistructured interview included questions related to their families' experiences with COVID-19. Sample questions included: “How fearful are you of your susceptibility to COVID-19?” and “How familiar are you with the Public Charge Rule?” During the interview, the lead researcher wrote down patterns or main ideas to create memos.

The lead researcher transcribed audio recordings of the interviews and then destroyed the transcripts after analysis. Data were analyzed based on the constant comparative method using Atlas.ti software to code the data.^25^ The constant comparative method is a core component of grounded theory, and it provides guidelines to construct a theory based on comparing incidents within collected data.^25,26^ Transcribed interviews were coded into analytic notes using line-by-line coding, which consisted of reading the transcripts to conceptualize main ideas. Afterward, the lead researcher used memos plus other analytic notes coded through Atlas.ai to engage in focused coding to separate, sort, and synthesize data. Finally, data were used to create a theory that provided an overall explanation of how mixed-status families faced social and health disparities during the COVID-19 pandemic.

## Results

Of the participants (*n*=14), 10 individuals identified with she/her/hers pronouns, 2 identified with he/him/his pronouns, and 2 identified with they/them/their pronouns. All participants lived within the Greater Los Angeles Region and were between the ages of 19 and 29 years. Table 1 summarizes that major interview themes with key quotations under each theme.

**Table 1.** Mixed-Status Family Interview Questions

Topic	Questions
General Introduction Questions about Volunteer Participants and their Families	To start, what is your age?
	Tell me about yourself, what city do you and your family live in?
	If you have one, tell me about your job.
	Do any of your other immediate family members work? If yes, what is their general occupation?
	Are you the household income producer (breadwinner)? If not, who is?
	You come from a mixed-status family, how is that like?
	What is your immigration status?
	If undocumented, are you on a path to naturalization?
	What is your immediate families' immigration status?
	If undocumented, are they on a path to naturalization?
	Tell me why you decided to take part in this study.
Mixed-Status Families' Experience with COVID-19	Tell me about you and your family's experience throughout the pandemic so far.
	How fearful are you of your susceptibility to COVID-19?
	Why?
	Why not?
	How familiar are you with the Public Charge Rule?
	If familiar, how has it affected you and your family?
	What was the biggest challenge your family faced throughout this current pandemic?
	What resources did you use to navigate that challenge?
	Did you draw upon government assistance?
	If yes, what kind?
	If no, why not?
	What do you think are some of the major issues mixed-status families face during the pandemic?
	How do you think the U.S. government is responding to those issues?

### Financial need

Every single interviewee disclosed some financial need in their mixed-status family from March 2020 to about a year later at the date of interview. While this varied from anticipated/minimal financial need to experiencing extreme financial need, all participants emphasized this challenge during their interview. As seen in the quotes in Table 2, financial need stemmed from work insecurity as many family members were essential workers earning a minimum wage. Half of the participants indicated that their families depended on a single main income producer to support the family throughout the pandemic. Financial need caused fear of not being able to afford rent, food, medical care, and/or immigration lawyer fees.

**Table 2.** Major Themes and Quotations from Participants Belonging to Mixed-Status Families

Theme	Selected Quotations
Financial Need	“My dad he is a business owner he fixes cars as I mentioned, but even though they were considered an essential business and stayed opened there was still like almost no clients coming through, so we went through a couple of months where it was really scary realizing what if we run out of money, what do we do then?”
	“Since it started like in March or April things got really bad and financially it's just been all over the place [with] worry about money just trying to make payments, and I know my dad and his wife had to refinance their house so they can continue to make payments on time and then, yeah, it's just been scary.”
	“Mostly kinda [what] took a big hit was our financials. My parents don't really make a stable income already and having COVID happen was a big…it was just really bad.”
	“Since the pandemic started…my mom was really affected. When the whole Covid happened she stopped making income, my dad as well, from like May [un]til September.”
Job Insecurity	“Both of my parents were not laid off, but they just stopped working [be]cause you know barber shops need to be closed, and my dad, even though he kind of works outside like it's still…they didn't have really good regulations. So, they stopped about March or May working or something, like I don't remember. It was like the beginning of the pandemic.”
	“That's when we got, all of our family members got Covid and so we all have to take a hiatus from work, aside from me because I work online.”
	“I guess economically they did stop working for like two months, like reduced hours and so that…it was affecting our mental health [be]cause we didn't know how we're gonna pay the rent and stuff like that.”
Housing Insecurity	“I think just the biggest challenge has been making sure we have a roof over our heads we just the worry was that we wouldn't be able to make rent payments on time.”
	“Rent is expensive and here in [this neighborhood in Los Angeles] it kinda gets worse and worse due to gentrification and we currently pay $1,300 for a small like 2 bedroom. It's also at the heart of [this neighborhood] so like it's kind of where the rents go up and…so we were worried about the rent and we used to pay $1,200, but then the landlord increased it by $100 during the pandemic [laughs to self]. So that was worse, and my parents did not take that very well so that was kinda bad.”
	“Financially it's hard to like pay for especially somewhere to live like an apartment or something. We're staying with our grandmother.”
Food Insecurity	“Food, it was but it kind [of] wasn't, because we buy things in bulk. So like when the pandemic started, we already had bulky things so we had [a] big [bag] of rice and beans. What we did start doing is rationing our food. So, like we cooked two cups of rice, now it's one cup of rice. We used one whole onion for the beans, now its half of an onion for the beans. So, we kind of started rationing our food for the first like three months of the pandemic.”
	“I think [my mother-in-law got] CalFresh for my youngest brother-in-law [who] was in high school, so that over the pandemic she was able to afford food.”
	“We're getting free food from I think the city had like a like a food pantry and then they would get food right there.”
Mental Health	“I was really anxious because like my family doesn't have a stable…home, so I didn't know exactly where I was going to go back [to] because I needed the Internet. So I just have a lot of anxiety from the pandemic…As a whole it also brought anxiety because since my dad no longer has a job he would have to apply for unemployment and it's hard to like sustain ourselves with unemployment.”
	“I will say in the mental situation…a little bit more stress from my side, I think, which is more exhaustion. Like, I was just so tired constantly. I…was just feeling like a rabbit where I would just get up, go to work, comeback.”
	“Our family was going through a lot at the time, too. Like, my grandma passed away during the pandemic, my parents were having problems, and I feel also the whole home school thing…I just don't. Everything I feel like affected my family…Yeah, like my brother is being at the house, too. I feel like all that…was just too much to unpack, you know. We never really been in a situation like that.”
Distrust of Government and Health Officials	“My mom…she's always scared of asking for other resources even if it says it's like confidential or that immigrant undocumented people or are okay. She usually doesn't really take that chance.”
	“Not fear of like, will things hurt you will things not hurt you, and not being able to take full advantage of things. Like, I know the city of [Los Angeles] was doing rent assistance and whatnot, and I know for sure we were just because like, how will it affect them or whatnot. Little things like that, just not knowing information.”
	“We don't have like any documentation…if we go to the hospital but it's going to happen…What will happen with that, with ICE? Are we? Are we going to be deported during this time? All of them [family members] were skeptical.”
Fear of Public Charge Rule	“I still felt that frustration and that stress knowing that that a little step or a little request or just even asking a question to someone who is connected with some federal aid or something it can mean that, you know, I am trying to be a burden to the government.”
	“We weren't able to have things like health insurance and…my parents were scared that if they asked for, like, government assistance that it would affect their application, so we've gone a long time. Well, I currently used [health] insurance from the university, but my siblings and my mom don't have health insurance. So that's something that was really concerning, especially now because of the pandemic, that we're really scared that we wouldn't be insured if something happened to us.”
	“I know that was one of the concerns we were talking about also is that that could potential[ly] affect in the future if my mom wanted permanent citizenship.”
	“I…told my mom about…applying to Calfresh and whatnot [be]cause she's a citizen now, so I was like you might as well take advantage of that or my little sister and stuff. They are fearful that in doing that my dad will get, like it'll affect his case…Even Covid test[ing], like taking them at the beginning [of the pandemic], [they] were [a] little cautious. What if it hurts his case and it makes him seem like he's leeching off of the government?”

Quotes have been edited slightly for readability.

ICE, U.S. Immigration and Customs Enforcement's.

Participants mentioned family members refinancing their homes, selling personal valuables, and/or taking out loans to be able to make payments for rent, food, and medical expenses. Mixed-status family members noted varying levels of financial need: extreme during only the beginning of the pandemic, prolonged throughout the pandemic, or at certain moments during the pandemic.

### Job insecurity

All participants similarly revealed that their families dealt with work insecurity. Participants mentioned how, due to the pandemic, they or their family members experienced job loss or reduction of hours from their jobs. Especially in the beginning of the pandemic, participants expressed how many of their family members were furloughed or received a reduction of hours due to the stay-at-home orders. Participants also expressed how if they or a family member were essential workers, they had instability in their work environment, especially if they became infected with COVID-19. For participants with family members who were at high-risk of mortality from COVID-19, some family members quit or left their jobs due to fear of exposure and infection. Interviewees or their family members who were undocumented experienced job loss from their “under the table” jobs and found it hard to find employment elsewhere.

### Housing insecurity

Of participants interviewed, the majority spoke about housing insecurity. Many participants addressed how their biggest challenge was to pay rent during the pandemic. They spoke about having to move during the pandemic, and/or moving in with other family members to be able to afford rent on time. Several of participants expressed having to live in overcrowded housing. Participants noted the high cost of rent in Southern California, and some even mentioned how their rent increased during the pandemic.

### Food insecurity

Participants spoke about food insecurity, not having enough money to buy food on their own during the pandemic. Members of mixed-status families depended in food banks/pantries for food. Some used resources such as federal Supplemental Nutrition Assistance Program (SNAP), known in California as CalFresh, for food. Volunteer participants also addressed how they also received food or money to buy food from extended family or community members during the pandemic.

### Mental health

Of all participants, many spoke about dealing with problems related to mental health. As seen in Table 2, participants spoke about how they and their family members endured heightened and extreme stress throughout the pandemic. When participants mentioned the death of a family member due to COVID-19, they expressed feelings of loss and depression. Due to the pandemic and threat of infection, participants expressed feelings of depression, anxiety, isolation, and panic. Participants addressed how these feelings of panic, stress, and anxiety were also closely tied to and exacerbated by the financial needs their families were facing during the pandemic.

### Distrust of government and health officials

Regarding distrust of government officials, the vast majority of participants spoke about this topic. Participants or their family members did not trust the resources that local and the California state government were providing for undocumented and mixed-status families during the pandemic. Mixed-status family members did not want to report that they or a family member were undocumented, so they preferred to not receive resources, even when they were suffering due to financial, job, housing, and food insecurity. This was also expressed in the form of distrust in health officials, noted by many of the participants. They expressed how they or their family members did not receive COVID-19 testing or treatment due to their fear of being exposed as undocumented.

Some participants even discussed how this distrust toward health officials created vaccine hesitancy. Volunteer participants and their families expressed how they did not want to be labeled as burdens to the government and therefore preferred not to receive any governmental help.

### Fear of public charge rule

Related to this distrust of government, participants spoke about their fear Public Charge Rule. Even when participants were not familiar with the term “Public Charge Rule,” they spoke about it with regard to their fear of being seen as a burden to the government, which would jeopardize any process of seeking legal citizenship. Some participants spoke about how they received benefits to survive, but they were always considering how it could affect their legal process of citizenship in the future. People who were undergoing the legal process of residency or citizenship were less likely to receive support from governmental resources or any nongovernmental resources (e.g., food banks) that could imply reliance on public aid. Some participants also spoke about how they did not have health insurance nor receive health care due to wanting to avoid receiving governmental benefits.

## Discussion

Due to job insecurity—job loss and/or a reduction of work hours—from their low-wage and often “essential” occupations, mixed-status families dealt with extreme financial need during the pandemic. This financial need caused housing and food insecurity for mixed-status families, which contributed to poor physical and mental health. Housing insecurity necessitated moving into overcrowded housing situations with family members to afford high rents, increasing susceptibility to COVID-19 infection. These insecurities contributed to worsened mental health among mixed-status family members, who struggled with anxiety, panic, and depression.

Many of these families needed resources to survive the COVID-19 pandemic but were afraid to receive any help due to distrust in the government and Public Charge Rule. Even if resources were highly publicized as not affecting Public Charge status, volunteer participants and their families distrusted the government, believing these resources were a trap made to expose them or their family members as undocumented under an anti-immigrant political regime.

While some volunteer participants and their families received support from sources such as food banks, governmental resources, and public health benefits, they still feared that using these resources could one day hurt them when they want to apply for their residency or citizenship. The majority of participants who accepted support received public nongovernmental resources instead of governmental resources. However, many participants and their families refused to accept help altogether as they did not trust government officials. This fear of the government expanded to distrust in health and medical officials, creating a situation in which families would not get tested for COVID-19 or go to the hospital for medical care if infected due to fear that the government might deport them or let them die in the hospital.

**FIG. 1.**
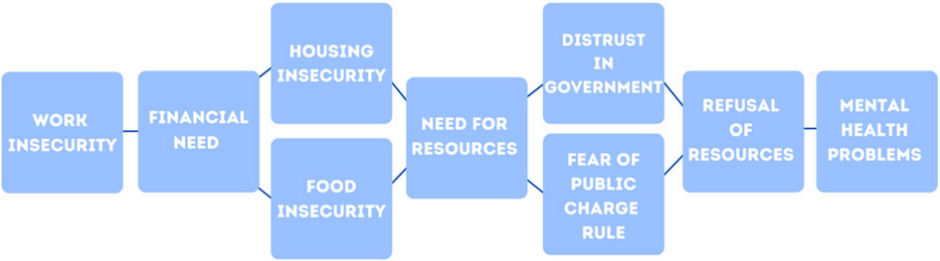
Framework for understanding lack of resources and mental health problems among members of mixed-status families during the COVID-19 pandemic.

Due to their financial need, they also could not afford routine medical care, so they avoided receiving any health care for mental or physical health due to cost. This is despite participants noting experiences with mental and physical health problems stemming from financial burden, death of family members, or being infected by COVID-19 themselves. Figure 1 provides a visual outline of the interconnectedness of each theme discussed previously in relationship to health disparities among members of mixed-status families.

Scholars predicted that Public Charge Rule would have a chilling effect on immigrant families receiving government and public benefits during the COVID-19 pandemic,^1,3,11^ and a few studies using quantitative data have suggested that this is the case among immigrant populations broadly.^13,14^ However, this is the first study to our knowledge to explore this phenomenon in-depth using data collected directly from members of the undocumented community and their families in the U.S. This study shows how mixed-status families navigated the precarious situation of COVID-19 while grappling with fears of retaliation from the government—whether in the form of immediate threats of deportation and detention, or future threats to the process of legalization or naturalization. Our findings highlight how these dual pandemics of COVID-19 and anti-immigrant sentiment resulted in food insecurity and poor mental health outcomes among members of mixed-status families.

### Strengths and limitations

It is typically difficult to recruit study participants who belong to a historically marginalized community, such as those who are part of the undocumented community and their family members. While this study made efforts to not collect or store identifiable information, there is nevertheless understandable hesitancy for participation due to fear that doing so would expose their own or their family members' undocumented statuses. Despite this challenge, the lead researcher was able to successfully recruit and interview a small but valuable sample of 14 participants from mixed-status families during the COVID-19 pandemic. As a bilingual and first-generation undergraduate student, the lead researcher was able to establish rapport and trust with interviewees, many of whom were themselves college students or young adults. Inherent biases likely exist in this sample, with those who were most hesitant not taking part. Middle- and older-age adults were not captured in the sample.

Furthermore, as recruitment and interviews took place virtually due to the nature of the COVID-19 pandemic, this study excluded individuals who did not have access to the Internet. Even so, this study provides valuable insights into the lives of mixed-status families from the perspectives of the young adult volunteer participants on navigating the difficult COVID-19 pandemic. Future research should consider capturing from an intersectionality perspective the effects of anti-immigration policies like Public Charge Rule on the experiences of people in mixed-status families at the intersections of compounding marginalized identities such as age, race, gender, immigration status, and socioeconomic status.^5,27,28^

## Health Equity Implications

The trust between mixed-status families and the government needs foundational rebuilding. Policies such as Public Charge Rule cause so much damage to the relationship between mixed-status families and the government that these families endure extreme and preventable suffering during a public health emergency such as the COVID-19 pandemic.^1,11^ As anti-immigration policies only cause fear and confusion among the most vulnerable, the government needs to work on reconstructing trust with mixed-status families to reduce health disparities and have more effective population health interventions.^15^ In addition to the social determinants of health previously mentioned, many individuals in this study expressed that they or family members had started the legal process to receive permanent residency or citizenship, but due to lawyer expenses and confusing policies were unable to complete the process.

One recommendation is to make the procedure to gain legal status in the U.S. for individuals within mixed-status families easier and more accessible. Doing so would enable more members of mixed-status families to gain permanent residency and citizenship, reducing health disparities.^29^ Finally, there is a need for specific policies that protect and support mixed-status families during public health emergencies. In moments of public health crisis such as the COVID-19 pandemic, mixed-status families must be protected through policy from extreme forms of financial need, housing instability, and food insecurity. Every individual in the U.S. deserves to have their basic need met during challenging times such as the COVID-19 pandemic, regardless of documentation status, especially as the pandemic has shown how interwoven our lives and livelihoods truly are.^30^

## Abbreviations Used

CARES: Coronavirus Aid, Relief, and Economic Security
ICE: U.S. Immigration and Customs Enforcement's
SNAP: Supplemental Nutrition Assistance Program
UCI: University of California, Irvine
U.S.: United States
